# Gastric and Small-Intestinal Morphological Remodeling After Intragastric Apelin-13 Administration in Unweaned Rats

**DOI:** 10.3390/ani16030497

**Published:** 2026-02-05

**Authors:** Sylwia Szymańczyk, Cezary Osiak-Wicha, Katarzyna Kras, Małgorzata Kapica, Iwona Puzio, Hanna Antushevich, Atsukazu Kuwahara, Ikuo Kato, Iwona Łuszczewska-Sierakowska, Marcin B. Arciszewski

**Affiliations:** 1 Department of Animal Physiology, Faculty of Veterinary Medicine, University of Life Sciences in Lublin, 20-950 Lublin, Polandiwona.puzio@up.edu.pl (I.P.); 2 Department of Animal Anatomy and Histology, Faculty of Veterinary Medicine, University of Life Sciences in Lublin, 20-950 Lublin, Polandmb.arciszewski@wp.pl (M.B.A.); 3 Department of Genetic Engineering, The Kielanowski Institute of Animal Physiology and Nutrition, Polish Academy of Sciences, 05-110 Jabłonna, Poland; 4 Laboratory of Physiology, Institute for Environmental Sciences, University of Shizuoka, 52-1 Yada, Suruga-ku, Shizuoka 422-8526, Japan; 5 Department of Psychosomatic Internal Medicine, Kagoshima University Graduate School of Medical and Dental Sciences, Kagoshima 890-8544, Japan; 6 Department of Correct Clinical and Imaging Anatomy, Faculty of Medical Sciences, Medical University of Lublin, 20-090 Lublin, Poland

**Keywords:** gastrointestinal maturation, enteric nervous system, mucosal remodeling, postnatal development

## Abstract

Apelin is a postnatal peptide implicated in gastrointestinal maturation, yet its combined effects on mucosa, enteric plexuses, and gut-derived appetite signals are not well defined. We investigated the impact of chronic intragastric apelin-13 on the stomach and small intestine of unweaned rats. Twelve Wistar pups of both sexes received apelin-13 (100 nmol/kg body weight, twice daily) or saline from postnatal day 10 for 14 days. After euthanasia, gastric and small-intestinal samples were processed for histomorphometry, neurofilament immunohistochemistry of myenteric and submucosal plexuses, and quantitative staining for ghrelin and leptin. Apelin-13 increased gastric mucosal thickness and pit and gland height, enlarged zymogen cells, and reduced muscularis propria thickness, while leaving submucosa and parietal cell area unchanged. In the small intestine, apelin produced a clear proximal-distal gradient, with enhanced villus-mucosa indices proximally and reduced indices in mid-to-distal jejunum, alongside broader crypt remodeling. Enterocyte and goblet cell dimensions changed in parallel with these regional shifts. Myenteric and submucosal ganglia were also remodeled in a segment-dependent manner. Ghrelin immunoreactivity increased in most regions, whereas leptin showed opposite proximal and distal responses. Overall, early-life luminal apelin-13 reshapes gastric and intestinal architecture and local hormone expression.

## Simple Summary

Young mammals rely on rapid growth of the stomach and intestines to transition from milk to solid food, but the hormones that guide this process are still being discovered. One such hormone is apelin, a protein naturally present in the digestive tract and in milk. We asked whether giving apelin directly into the stomach of newborn rats would change how their stomach and small intestine develop. Ten-day-old rat pups received apelin or placebo for two weeks, after which we examined the tissues and measured the thickness of the gut wall, the size of surface cells that absorb nutrients, the mucus-producing cells that protect the lining, and the structure of nerve networks inside the gut that control movement. We also measured two appetite-related hormones made in the gut, ghrelin, and leptin. Apelin made the stomach lining thicker and changed some digestive cells, and it reshaped the small intestine depending on the region, with stronger effects in the upper part. The gut nerve networks and local ghrelin and leptin signals also changed. These findings show that apelin can influence early digestive development and may help explain how feeding hormones program gut function in infancy, which could be useful for improving neonatal nutrition.

## 1. Introduction

Apelin-13 is an endogenous peptide belonging to the apelin peptide family, which was first identified by Tatemoto et al. [1]. It is derived from the precursor protein preproapelin and represents one of several isoforms differing in amino-acid length [2]. Among these isoforms, apelin-13 exhibits the highest affinity and potency toward the APJ receptor [2]. The apelin-APJ system shows a broad tissue distribution in mammals, with particularly high expression reported in the central nervous system (CNS), mammary gland, and lung [3,4]. Apelin/APJ signaling is present in multiple physiological systems and has been investigated as a therapeutic target in cardiovascular diseases [5], respiratory disorders [6], neurological conditions [7], kidney diseases [8], metabolic disorders [9], musculoskeletal diseases [10], cancer [11], and gastrointestinal pathologies [12], including liver disorders [13]. This diversity highlights the functional versatility of the apelinergic pathway.

Although the apelin system is widely expressed across organs, its role in the gastrointestinal tract is of particular interest. The intestine functions as a major endocrine organ, and apelin produced by enterocytes and enteroendocrine cells has been implicated in the regulation of epithelial proliferation, gastrointestinal motility, glucose absorption, and mucosal barrier integrity [14,15,16]. Apelin mRNA is abundant in the stomach and also detectable in the small intestine [16]. Immunohistochemical studies further show that apelin is abundantly expressed in the gastric mucosa, particularly within mucous neck, parietal, chief (zymogen), and specific subpopulations of chromogranin A- positive enteroendocrine cells, while it is absent from the muscular layer [17]. APJ is most prominently expressed in the gastric fundic mucosa and in the proximal small intestine, particularly within the villus epithelium, supporting the concept that apelin participates in local hormonal and paracrine regulation of digestive function [12,18].

Evidence suggests that apelin may play an important role in the postnatal development of the gastrointestinal tract. In young rats, exogenous apelin-13 influences the structural maturation of the stomach and intestines and alters mucosal growth indices, indicating that the apelinergic system may participate in shaping early gastrointestinal architecture [19]. Beyond its effects on epithelial dynamics, apelin interacts with central and peripheral neural mechanisms that regulate digestive function [20,21,22]. APJ receptor is expressed in gut-associated neural structures, including enteric neurons and vagal afferent pathways, and apelin may modulate neural circuits that regulate gastrointestinal motility and nutrient handling [20]. However, the effects of chronic early-life apelin exposure during early life on enteric nervous system (ENS) structure or maturation have not yet been established. Moreover, apelin is also linked to the regulation of gut-derived endocrine signals. Administration of apelin-13 has been shown to influence circulating ghrelin and leptin levels and modifies appetite-related pathways [23].

Taken together, current evidence suggests that apelin can modulate gastrointestinal structure, neural pathways, and endocrine signals. However, its integrated effects on histomorphology, ENS organization, and gut hormone expression during early postnatal development remain poorly characterized. We hypothesize that early-life apelin exposure alters structural maturation of the stomach and intestines, remodels the ENS, and modifies local ghrelin and leptin signaling, thereby contributing to long-term programming of digestive and metabolic function. Therefore, the present study examined gastric and intestinal architecture, neurofilament, ghrelin, and leptin immunoexpression in young rats treated with apelin-13.

## 2. Materials and Methods

### 2.1. Animals and Study Design

The study was conducted on twelve unweaned Wistar rats of both sexes (body weight 20–25 g), with all procedures approved by the II Local Ethics Committee for Experiments on Animals in Lublin, Poland (approval no. 49/2008), that were equally distributed between the control and apelin groups, with two offspring from each dam assigned to each group. The study was not powered for sex-stratified analysis, and data from males and females were therefore pooled for all outcome measures. Pregnant dams were purchased from Breeding of Laboratory Animals, Brwinów, Poland and housed under standard husbandry conditions in the animal facility of the University of Life Sciences in Lublin (Poland). From birth until the end of the experiment, the pups remained with their dams under standard housing conditions (temperature 21 ± 1 °C, relative humidity 60–70%, 12 h light/dark cycle). On postnatal day 10, the offspring were individually marked and randomly assigned to either the control group or the apelin-treated group, with six animals per group (*n* = 6). To minimize litter-related bias, two pups from each dam were allocated to each group, ensuring comparable maternal background and early exposure to apelin derived from breast milk.

The experimental group received apelin-13 (Hokuriku University, Kanazawa, Japan) intragastrically via a flexible cannula designed for small rodents (AgnTho’s, Lidingö, Sweden). The peptide was administered at a dose of 100 nmol/kg body weight twice daily (at 09:00 and 21:00) for 14 consecutive days. The control group was treated in parallel with an equivalent volume of physiological saline using the same intragastric procedure. The dose of apelin-13 was chosen based on previous studies in rats [24].

### 2.2. Sample Processing

After 14 days of treatment, approximately 12 h after the last apelin-13 administration, all rats were weighed and euthanized by carbon dioxide inhalation. As the animals were non-weaned and remained with their mothers throughout the experiment, they were not subjected to pre-euthanasia fasting in order to avoid additional stress and metabolic disturbance. A midline laparotomy was then performed, and the stomach and small intestine were gently removed from each animal, freed from surrounding tissues, carefully blotted dry, and weighed. The small intestine was straightened without tension and its total length was measured. From each rat, five full-thickness segments of approximately 1 cm were collected in a standardized manner as follows: from the duodenum immediately below the pyloroduodenal junction; from the jejunum at 25% (proximal), 50% (middle), and 75% (distal) of the total small intestinal length; and from the ileum located 2 cm proximally to the ileocecal junction. The stomach was dissected along the greater curvature, rinsed gently with physiological saline to remove residual contents, blotted dry, and weighed. Tissue samples were then dissected from the fundic and pyloric regions for further microscopic analyses.

All tissues were immediately immersed in 4% phosphate-buffered formaldehyde (pH 7.0) and fixed for 24 h at room temperature. Following fixation, the material was dehydrated in a graded series of ethanol solutions, cleared using Ottix Shaper and Ottix Plus reagents (DiaPath, Martinengo, Italy), and processed in an STP 120 spin tissue processor (Waltham, MA, USA) before paraffin embedding. Paraffin blocks were sectioned at a thickness of 4 µm with a Microm HM 360 rotary microtome (Microm, Walldorf, Germany). The sections were mounted on glass slides, including adhesive slides intended for immunohistochemistry, dried, and prepared for routine histological staining, morphometric examination of the gastric and small intestinal wall and the myenteric (MP) and submucosal plexuses (SP), as well as for immunohistochemical assessment of ghrelin and leptin immunoexpression.

### 2.3. Histology and Morphometric Analysis

Paraffin sections of the duodenum, jejunum, ileum, and stomach were stained with haematoxylin and eosin (H&E) for general histological evaluation and basic morphometry. In addition, sections from the gastric fundus and pylorus, as well as from all small intestinal segments, were stained using a combined Alcian Blue (pH 2.5) and Periodic Acid-Schiff (AB-PAS) method in order to count mucus-secreting cells and assess their distribution.

Microscopic observations and morphometric measurements were performed using an Olympus BX63 light microscope equipped with a digital camera and computer-assisted image analysis software (cellSens, Version 1.5; Olympus, Tokyo, Japan). Images were recorded at objective magnifications of 4×, 10×, 20×, and 40× to document overall architecture and to obtain high-resolution fields for quantitative analysis.

For each animal, one well-oriented section from each small intestinal segment (duodenum; three jejunal levels at approximately 25%, 50%, and 75% of total length; and ileum) was selected, with villi and crypts cut perpendicular to the mucosal surface. In H&E- and AB-PAS-stained sections, the following parameters were measured: mucosal and submucosal thickness, villus height, crypt depth, villus height-to-crypt depth ratio, thickness of the tunica muscularis, mean enterocyte area, and the number of AB-positive and PAS-positive goblet cells. For each segment, ten well-aligned villi with their corresponding crypts were analyzed. Mucosal thickness was measured from the base of the crypts to the tips of the corresponding villi. Villus height was defined as the distance from the villus tip to the level of the crypt opening, whereas crypt depth was measured from the base of the crypt to the line connecting the bases of adjacent villi. The villus-to-crypt ratio was calculated by dividing mean villus height by mean crypt depth. Enterocyte area was determined in the mid-portion of well-oriented villi by measuring the profile area of 20 enterocytes per villus. Goblet cells were counted along the length of ten villi in each segment in AB-stained sections and expressed as the number of AB-positive goblet cells per villus-crypt complex. Cell density (cells/mm^2^) was determined by delineating a defined mucosal area (mm^2^) in ImageJ 1.53 (National Institutes of Health, Bethesda, MD, USA) https://imagej.net/ij/index.html (accessed on 28 May 2025), counting the number of cells within this region using the cell counter tool, and expressing the result as cell count divided by the measured area. All measurements were performed in micrometers or micrometers squared using the calibrated image analysis system.

In gastric fundic sections, only preparations in which the pits and glands were cut perpendicular to the mucosal surface were used for morphometry. The following parameters were assessed: thickness of the gastric mucosa, height of the gastric pits, thickness of the submucosa, and thickness of the muscularis propria. Parietal and chief cells were evaluated in the corpus mucosa. For each cell type, the mean cross-sectional area and the number of cells per unit area were determined in standardized regions of interest extending from the luminal surface to the muscularis mucosae.

### 2.4. Immunohistochemical Staining and Evaluation

Immunohistochemical detection of neurofilament, ghrelin, and leptin was carried out on paraffin sections of the duodenum, jejunum, ileum, gastric fundus, and pylorus, according to standard protocols for paraffin-embedded tissue. Neurofilament staining was used to visualize the MP and SP, whereas ghrelin- and leptin-immunoreactive cells were assessed mainly within the gastric and intestinal mucosa.

Sections were deparaffinized in xylene, rehydrated through graded ethanol, and subjected to heat-induced antigen retrieval by boiling in citrate buffer (pH 6.0) for 8 min in a microwave oven (700 W). Endogenous peroxidase activity was blocked with 3% hydrogen peroxide for 10 min at room temperature. Non-specific protein binding was then reduced by incubation with UltraVision Protein Block (Thermo Fisher Scientific, Waltham, MA, USA) in accordance with the manufacturer’s instructions. After blocking, sections were incubated with the primary antibodies listed in Table 1 for 30 min at room temperature. This step was followed by a 30 min incubation with BrightVision Poly-HRP anti-mouse/rabbit IgG, biotin-free, one-component ready-to-use reagent (ImmunoLogic, Duiven, The Netherlands). The peroxidase reaction was developed with 3,3′-diaminobenzidine (DAB; UltraVision Plus Detection System, RTU; Thermo Fisher Scientific, Waltham, MA, USA) for 5 min at room temperature. All slides were then rinsed, counterstained with Mayer’s haematoxylin, dehydrated, cleared, and coverslipped. Negative controls were prepared in parallel by omitting the primary antibody while keeping all other steps identical.

Microscopic evaluation and image acquisition were performed using the same Olympus BX63 light microscope equipped with a digital camera and cellSens image analysis software (Version 1.5; Olympus, Tokyo, Japan) as described for the histological analysis. For quantitative assessment of ghrelin and leptin expression, representative fields were captured at standard magnifications. In each field, the reference tissue area (intestinal or gastric expressed as %) and the area occupied by DAB-positive structures (µm^2^/mm^2^) were measured with a pixel counting approach using the color threshold function of the software. This function allowed precise isolation of immunopositive areas based on color and hue, which facilitated evaluation of the spatial distribution of the reaction. The relative amount of immunoreactivity was then calculated as the area of DAB-positive signal in relation to the corresponding tissue area. For ghrelin and leptin, the outcome measure was the percentage of DAB-positive mucosal area per reference mucosal area, obtained by threshold-based segmentation of immunoreactive pixels. This parameter reflects the overall density of immunoreactive labelling and does not distinguish between changes in the number of positive cells and changes in immunoreactivity per cell.

### 2.5. Statistical Analysis

All statistical analyses were performed using GraphPad Prism version 10.6.1 for Windows (GraphPad Software, San Diego, CA, USA) https://www.graphpad.com/ (accessed on 9 May 2025). For each morphological and immunohistochemical parameter of the small intestine, the individual rat was treated as the experimental unit; measurements obtained from multiple villi or microscopic fields within a given segment were averaged to yield one mean value per segment per animal.

Data distribution was assessed using the Shapiro–Wilk test, and homogeneity of variances was evaluated with Levene’s test. Variables that met the assumptions of normality and homoscedasticity were analyzed using a two-way repeated-measure ANOVA with treatment (control vs. apelin) as a between-subjects factor and intestinal segment (duodenum; jejunum at 25%, 50% and 75% of total length; ileum) as a within-subjects factor. The model included main effects of treatment and segment as well as the treatment × segment interaction. The global F-tests for the main effects and interaction were used to assess the following: overall differences between control and apelin groups, physiological variation along the small intestine, and whether the effect of apelin depended on the intestinal segment. When the two-way ANOVA indicated a significant treatment × segment interaction or a significant treatment main effect, post hoc multiple comparisons were carried out using Tukey’s test. In the context of the present study, the primary contrasts of interest were the pairwise comparisons between control and apelin-treated rats within each intestinal segment; between-segment comparisons were considered secondary and used only to aid physiological interpretation of the results. For gastric morphometric and immunohistochemical parameters, comparisons between the control and apelin-treated groups were performed using an unpaired two-tailed Student’s *t*-test. All results are presented as mean ± standard error of the mean (SEM). A *p*-value < 0.05 was considered statistically significant for all tests.

## 3. Results

### 3.1. Stomach Morphology

**Figure 1:**
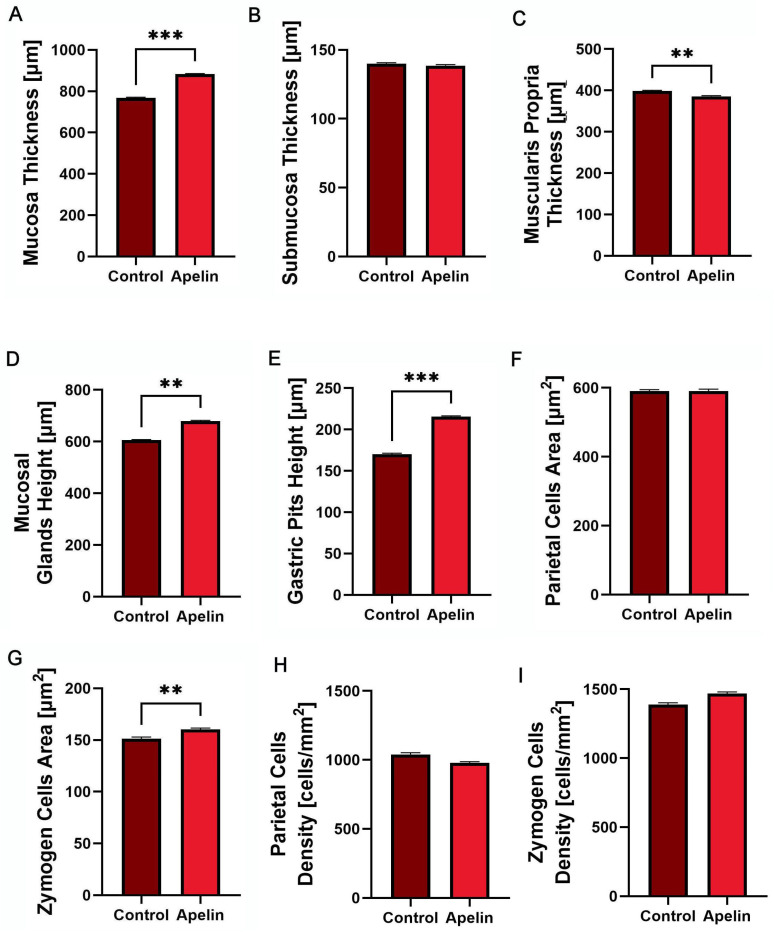
This Effects of apelin-13 on stomach wall morphology and epithelial cell size in unweaned Wistar rats. (**A**) Mucosa thickness, (**B**) submucosa thickness, (**C**) muscularis propria thickness, (**D**) mucosal gland height, (**E**) gastric pit height, (**F**) parietal cell cross-sectional area, (**G**) chief (zymogen) cell cross-sectional area, (**H**) parietal cell density, and (**I**) zymogen cell density in control and apelin-treated groups. Data are presented as means ± SEM (*n* = 6 per group). Asterisks (*) over the brackets indicate the significance of the difference between control and apelin groups (** *p* < 0.01, *** *p* < 0.001).

**Figure 2:**
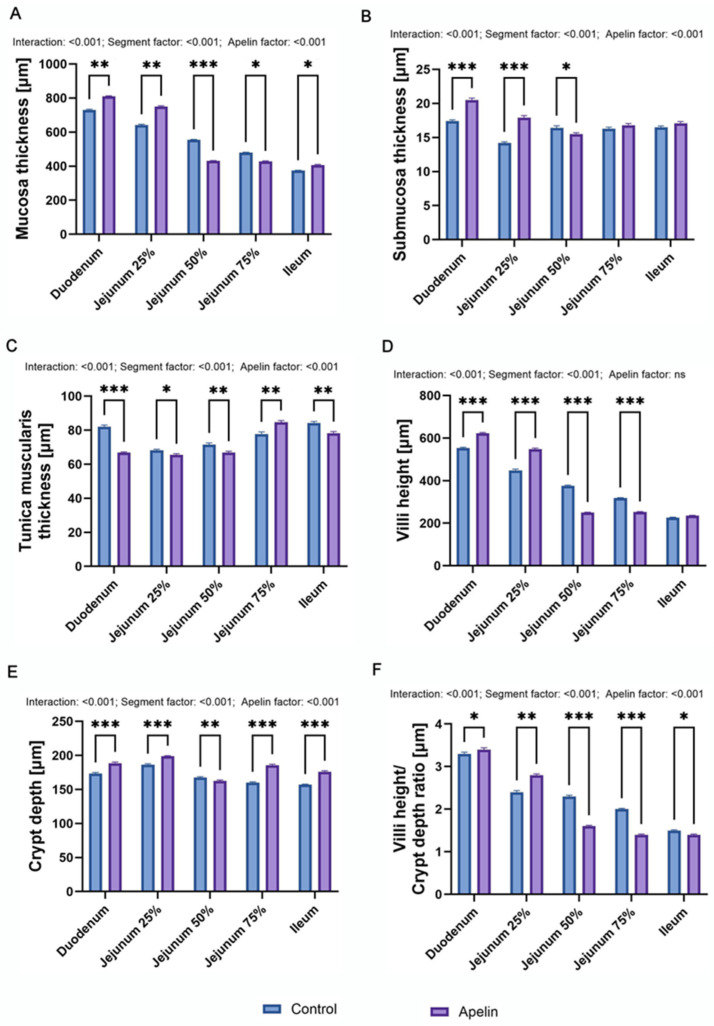
Effects of apelin-13 on intestinal wall morphology in unweaned Wistar rats. (**A**) Mucosa thickness, (**B**) submucosa thickness, (**C**) tunica muscularis, (**D**) villi height, (**E**) crypt depth, (**F**) villi-height-to-crypt-depth ratio in control and apelin-treated groups. Data are presented as means ± SEM (*n* = 6 per group). Asterisks (*) over the brackets indicate the significance of the difference between control and apelin groups (* *p* < 0.05, ** *p* < 0.01, *** *p* < 0.001). Numerical values placed above the graphs denote *p*-values for the interaction term and the main effects of the factors included in the two-way ANOVA model).

Apelin-13 administration caused a clear thickening of the stomach mucosa compared with the controls (Figure 1A; *p* < 0.001), whereas the thickness of the submucosa did not differ between groups (Figure 2B). In contrast, the muscularis propria was thinner in the apelin-treated rats than in the control animals (Figure 2C; *p* < 0.01). Within the mucosa, both the height of the stomach glands and the height of the gastric pits were greater in the apelin group (Figure 1D,E; *p* < 0.01 and *p* < 0.001 accordingly). The mean cross-sectional area of parietal cells remained unchanged (Figure 1F), while the area of zymogen cells was increased in the apelin-treated rats (Figure 1G; *p* < 0.01). There were no changes in cell density (Figure 1H,I).

### 3.2. Intestinal Morphology

Two-way ANOVA with segment and treatment as factors showed a significant interaction and significant main effects for segment and apelin for all the analyzed variables, with the exception of villus height, where only the segment effect was significant (all *p* < 0.001; F). Mucosal thickness differed between segments and between groups in a segment-dependent manner (Figure 2A). In the apelin group, mucosa was thicker in the duodenum and in the proximal jejunum (F (4, 50) = 401.2; 25% of total length; *p* < 0.01 vs. control), whereas it was reduced in the middle and distal jejunum (50% and 75%; *p* < 0.001 and *p* < 0.05 accordingly). In the ileum, the apelin-treated rats showed a slight increase in mucosal thickness compared with the controls (*p* < 0.05). Submucosal thickness (F (4, 50) = 30.66; Figure 2B) was greater in the apelin group in the duodenum and proximal jejunum (*p* < 0.001), lower in the middle jejunum (*p* < 0.05), and did not differ between groups in the distal jejunum and ileum. The tunica muscularis (F (4, 50) = 39.12; Figure 2C) was generally thinner in the apelin-treated rats. A significant decrease was observed in the duodenum, middle jejunum, distal jejunum, and ileum, with a milder reduction in the proximal jejunum (*p* < 0.05). In contrast, at 75% of jejunal length, the tunica muscularis was thicker in the apelin group than in controls (*p* < 0.01). Villus height (F (4, 50) = 443.6; Figure 2D) was higher in the duodenum and proximal jejunum in the apelin-treated rats (*p* < 0.001), and lower in the middle and distal jejunum (*p* < 0.001), with no difference between the groups in the ileum. Crypt depth (F (4, 50) = 38.50; Figure 2E) increased in most segments in the apelin group (duodenum, proximal and distal jejunum, ileum; *p* < 0.001), whereas a modest decrease was noted in the middle jejunum (*p* < 0.01). The villus height-to-crypt depth ratio (F (4, 50) = 142.1; Figure 2F) was higher in the duodenum (*p* < 0.05) and proximal jejunum (*p* < 0.01) of the apelin-treated rats but was reduced in the middle and distal jejunum (*p* < 0.001). In the ileum, the ratio was also slightly lower in the apelin group (*p* < 0.05)

### 3.3. Enterocyte and Goblet Cell Morphology

**Figure 3:**
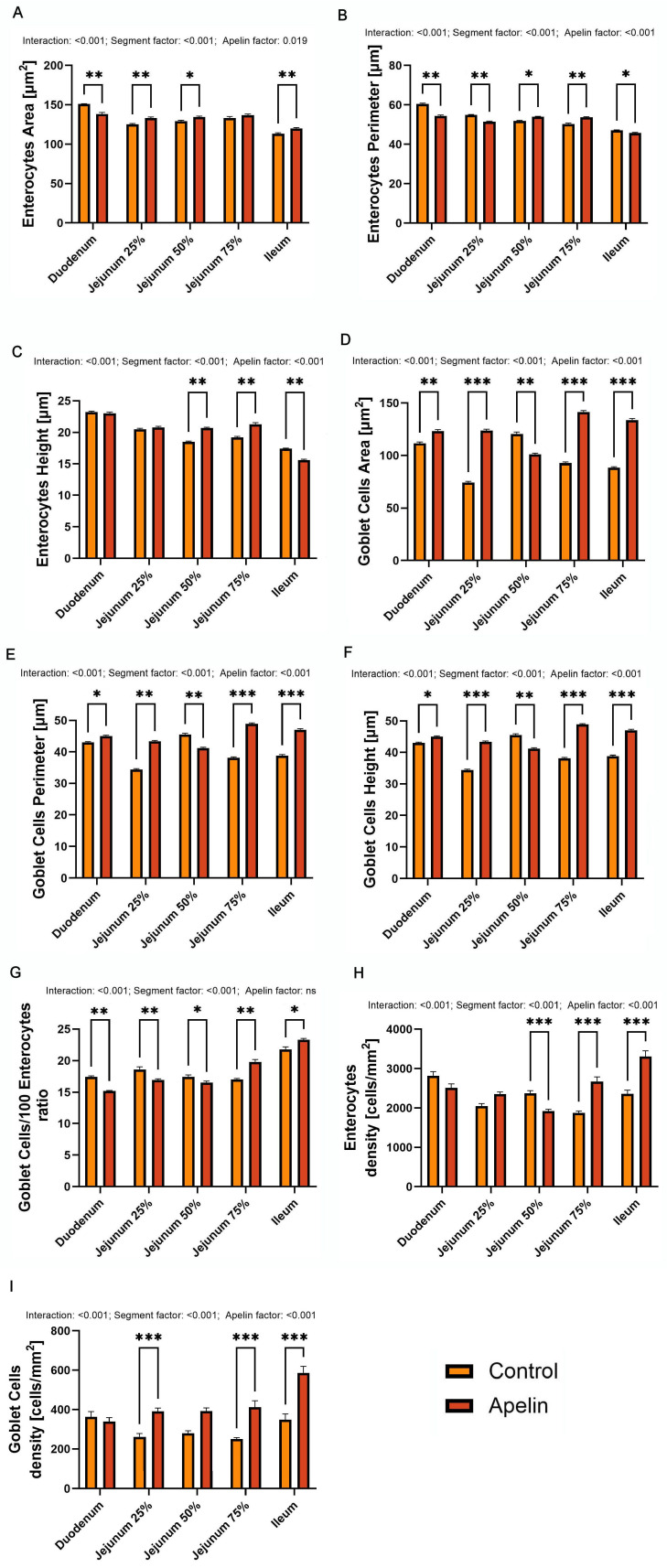
Effects of apelin-13 on enterocyte and goblet cell morphology in unweaned Wistar rats. (**A**) enterocyte area, (**B**) enterocyte perimeter, (**C**) enterocyte height, (**D**) goblet cell area, (**E**) goblet cell perimeter, (**F**) goblet cell height, (**G**) goblet cells to 100 enterocytes ratio, (**H**) enterocyte density, and (**I**) goblet cell density in control and apelin-treated groups. Data are presented as means ± SEM (*n* = 6 per group). Asterisks (*) over the brackets indicate the significance of the difference between control and apelin groups (* *p* < 0.05, ** *p* < 0.01, *** *p* < 0.001). Numerical values placed above the graphs denote *p*-values for the interaction term and the main effects of the factors included in the two-way ANOVA model.

Two-way ANOVA with segment and treatment as factors showed a significant interaction and significant main effects of segment and apelin for all analyzed variables related to enterocyte and goblet cell morphology, with the exception of the goblet cell-to-enterocyte ratio, for which the main effect of apelin was not significant. For the enterocyte area, the main effect of the apelin reached significance (*p* = 0.019), while all other interaction and segment effects were highly significant (*p* < 0.001). Enterocyte area (F (1, 50) = 5.87; Figure 3A) in the apelin group was lower in the duodenum than in the controls (*p* < 0.01), but higher in the proximal jejunum (*p* < 0.01), middle jejunum (*p* < 0.05), and ileum (*p* < 0.01). In the distal jejunum (75% of total length), enterocyte area did not differ between groups. Enterocyte perimeter (F (4, 50) = 38.29; Figure 3B) was increased in the middle and distal jejunum in the apelin-treated rats (*p* < 0.05 and *p* < 0.01 accordingly), whereas it was reduced in the duodenum, proximal jejunum, and ileum (*p* < 0.01 for all, except *p* < 0.05 in the ileum). Enterocyte height (F (4, 50) = 58.28; Figure 3C) was greater in the middle and distal jejunum of the apelin group compared with the controls (*p* < 0.01), lower in the ileum (*p* < 0.01), and unchanged in the duodenum and proximal jejunum. Goblet cell area (F (4, 50) = 262.9; Figure 3D) was significantly increased in most segments in the apelin-treated rats (*p* < 0.001), with only a slight reduction in the middle jejunum compared with the controls. Goblet cell perimeter and height (F (4, 50) = 209.5; perimeter and F (4, 50) = 226.4 height; Figure 3E,F) showed the same segmental pattern as the goblet cell area. The number of goblet cells per 100 enterocytes (F (4, 50) = 29.84; Figure 3G) was lower in the duodenum (*p* < 0.001), proximal jejunum (*p* < 0.001), and middle jejunum (*p* < 0.05) in the apelin group, but was higher in the distal jejunum and ileum (*p* < 0.001 for both). Enterocyte density (F (4, 50) = 245.6; Figure 3H) was significantly reduced in the middle jejunum and increased in the distal jejunum and ileum of the apelin-treated rats compared with thecontrols (*p* < 0.001 for all). Goblet cell density (F (4, 50) = 201.7; Figure 3I) was higher in the proximal and distal jejunum and in the ileum in the apelin group.

### 3.4. Enteric Plexus Morphometry

**Figure 4:**
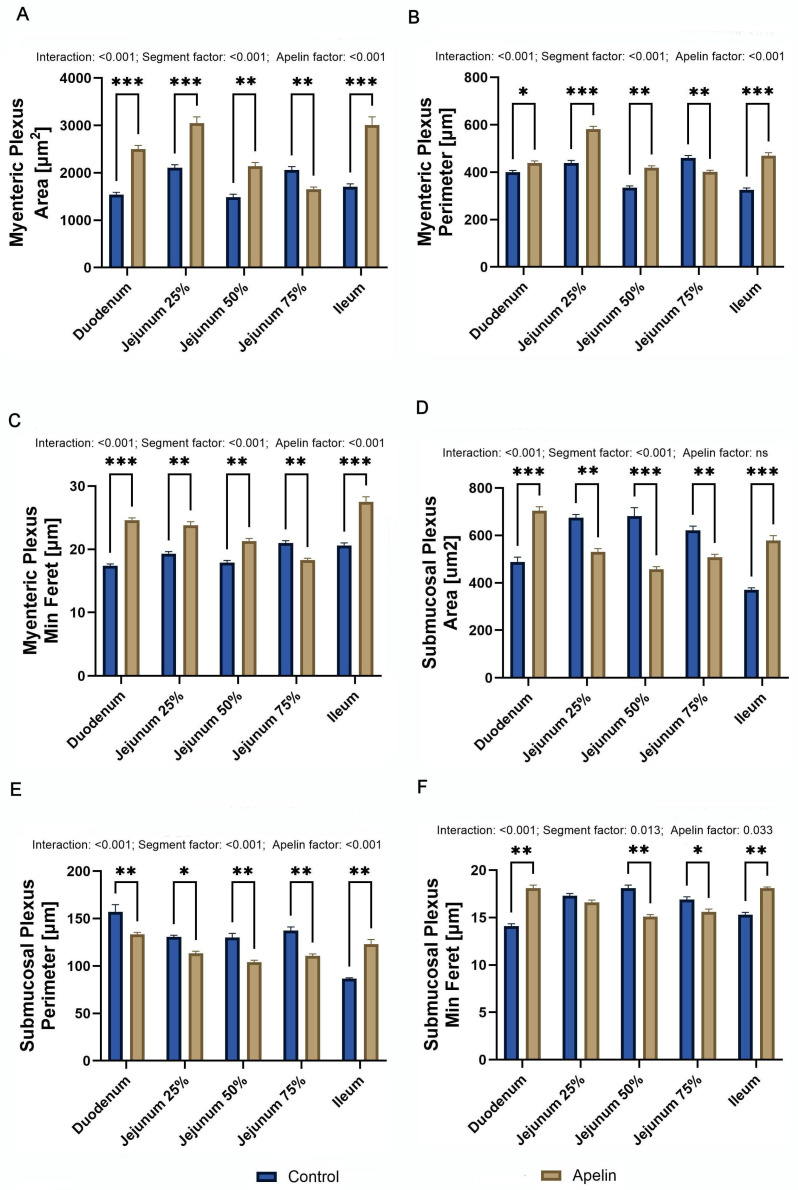
Effects of apelin-13 on the enteric plexus morphology in unweaned Wistar rats. (**A**) myenteric plexus area, (**B**) myenteric plexus perimeter, (**C**) myenteric plexus minimum Feret diameter, (**D**) submucosal plexus area, (**E**) submucosal plexus perimeter, (**F**) submucosal plexus minimum Feret diameter in control and apelin-treated groups. Data are presented as means ± SEM (*n* = 6 per group). Asterisks (*) over the brackets indicate the significance of the difference between control and apelin groups (* *p* < 0.05, ** *p* < 0.01, *** *p* < 0.001). Numerical values placed above the graphs denote *p*-values for the interaction term and the main effects of the factors included in the two-way ANOVA model.

Two-way ANOVA with segment and treatment as factors showed a significant interaction and significant main effects of segment and apelin for all MP and SP variables, with the exception of SP area, where the main effect of apelin was not significant. For all parameters, *p* < 0.001, except for SP min Feret diameter, where the segment effect was weaker (F (1, 50) = 4.81; *p* = 0.013) and the apelin effect reached *p* = 0.033. MP area (Figure 4A) was greater in the apelin-treated rats than in the controls in the duodenum, proximal jejunum, middle jejunum, and ileum (*p* < 0.001 for all except middle jejunum *p* < 0.01), whereas in the distal jejunum the MP area was reduced (*p* < 0.01). MP perimeter (F (4, 50) = 38.29; Figure 4B) followed the same pattern as area, with higher values in the duodenum, proximal and middle jejunum, and ileum (*p* < 0.001; middle and distal jejunum *p* < 0.01, duodenum *p* < 0.05) and lower values in the distal jejunum. MP min Feret diameter (F (4, 50) = 40.57; Figure 4C) also showed an increase in all segments in the apelin group compared with the controls (*p* < 0.001 duodenum and ileum, *p* < 0.01 jejunum). For the SP, SP area (F (4, 50) = 63.80; Figure 4D) was increased in the duodenum and ileum in the apelin-treated rats (*p* < 0.001 and *p* < 0.01 respectively) and decreased in the proximal, middle, and distal jejunum (*p* < 0.01 middle, *p* < 0.001 rest). SP perimeter (F (4, 50) = 26.75; Figure 4E) was lower in the duodenum, proximal, middle, and distal jejunum in the apelin group (*p* < 0.01; in the proximal jejunum *p* < 0.05), but was higher in the ileum (*p* < 0.01). SP min Feret diameter (F (4, 50) = 63.81; Figure 4F) was increased in the duodenum and ileum (*p* < 0.01), decreased in the middle and distal jejunum (*p* < 0.01 and *p* < 0.05, respectively), and did not differ between groups in the proximal jejunum.

### 3.5. Ghrelin and Leptin Immunoreactivity

Two-way ANOVA with segment and treatment as factors showed a significant interaction and significant main effects of segment and apelin for all parameters related to ghrelin and leptin percentage (F (5, 60) = 44.70 for ghrelin and F (4, 50) = 23.90 for leptin; *p* < 0.001 for interaction and both main factors). Ghrelin percentage was higher in the apelin-treated rats than in the controls in all examined segments (stomach, duodenum, proximal jejunum, distal jejunum, and ileum; *p* < 0.001), with the exception of the middle jejunum, where no difference between groups was found. Leptin percentage increased in the stomach (*p* < 0.001) and in the distal jejunum (*p* < 0.05), decreased in the duodenum and ileum (*p* < 0.001), and did not differ between groups in the proximal and middle jejunum. All quantitative data for ghrelin and leptin are summarized in Table 2.

## 4. Discussion

The present study investigated how chronic intragastric administration of apelin-13 affects the developing stomach and small intestine of unweaned rats. We focused on structural remodeling of the gastric and intestinal wall, quantitative changes in enterocytes and goblet cells, morphology of the myenteric and submucosal plexuses, and the immunohistochemical expression of ghrelin and leptin along the stomach–intestine axis. The data show that apelin-13 modifies several layers of the gastrointestinal wall in a segment-dependent manner and is associated with marked changes in orexigenic and anorexigenic peptide expression.

Our gastric morphometric data are consistent with apelin acting mainly on the mucosal compartment of the stomach. Previous studies have shown strong apelin immunoreactivity in mucous neck, parietal, and chief cells of the rat oxyntic mucosa, with little or no staining in the muscle layer, suggesting that gastric epithelial cells are the principal targets of apelin. [17]. Antushevich et al. reported that intragastric or intraperitoneal apelin in young rats modulates epithelial apoptosis and mitosis and influences the expression of the DNA repair enzyme OGG1/2, which they interpreted as an effect on mucosal maturation and cytoprotection [19]. In this context, the thicker mucosa and taller glands and pits that we observed after intragastric apelin-13 may reflect altered epithelial turnover or differentiation rather than simple cell swelling, although we did not measure proliferation or apoptosis markers and this interpretation remains speculative. The increase in chief cell area without a change in parietal cell size is consistent with the view that apelin is involved in the regulation of gastric secretory cell populations. Transcriptomic data indicate that apelin is expressed in parietal cells and can inhibit acid secretion through reduced histamine release from enterochromaffin-like cells [25], while functional studies in normal and diabetic rats suggest that repeated apelin-13 administration has limited impact on acid output in healthy animals but can improve gastric acidity and mucin content under pathological conditions [26]. Together with reports that apelin expression increases in the gastric mucosa during stress-induced injury and contributes to mucosal protection [27], our findings support the idea that apelin-13 participates in structural adaptation of the gastric mucosa in early life, which may have functional consequences that remain to be tested. The reduction in muscularis propria thickness that accompanied mucosal changes is in line with studies showing that both central and peripheral apelin-13 can inhibit gastric motility [21], although in the present work we did not assess motor function, so any link between muscle thinning and motility remains hypothetical.

The segment-dependent remodeling that we observed in the small intestine, together with the changes in enterocyte and goblet cell morphology, suggests that apelin-13 is associated with region-specific changes in the crypt–villus axis. Ontogeny studies in rodents have shown that apelin and, in particular, its receptor APJ are highly expressed around birth and in early postnatal life in the gastrointestinal tract, with robust APJ immunostaining in the intestinal surface epithelium, goblet cells, and smooth muscle, especially in the duodenum, which fits well with the more pronounced structural response that we recorded in the duodenum and proximal jejunum [19,28]. Intragastric apelin-13 in young rats has been reported to reduce apoptosis and modify mitosis in the duodenum and jejunum and to alter OGG1/2 expression, while in vitro apelin changes apoptosis and proliferation in crypt-derived intestinal cell lines [19,29]. These data, together with our finding of increased mucosal thickness, higher villi, and a higher villus-to-crypt ratio in the proximal small intestine, are consistent with a trophic influence of apelin on the immature mucosa, although we did not measure proliferation or DNA repair markers and therefore this interpretation remains hypothetical. Recent work has extended the role of apelin in epithelial biology by showing that exercise-induced apelin-APJ signaling enhances duodenal villus and crypt growth and increases Muc2 expression, a goblet cell marker, through AMPK activation, and that maternal exercise improves fetal intestinal epithelial development in an apelin-dependent manner [30]. These findings are in agreement with the enlargement of goblet cells and the rise in goblet-cell-to-enterocyte ratio that we observed in proximal segments. In the middle and distal jejunum and in the ileum, apelin-13 was associated with deeper crypts, a lower villus-to-crypt ratio, and only limited changes in villus height, together with a mixed pattern of enterocyte size and goblet cell abundance. This proximal–distal divergence may relate to the intestinal glucose–apelin cycle, in which apelin produced by proximal enterocytes responds to luminal glucose and regulates carbohydrate absorption through AMPK-dependent modulation of SGLT1 and GLUT2 expression, since this mechanism is most active in the upper small intestine [15,30], and it is tempting to speculate that similar mechanisms contribute here, although our study did not investigate nutrient transport or signaling pathways. From a functional point of view, goblet cells and the mucus they produce are key elements of the intestinal barrier and mucosal immunity, and developmental studies show marked age-related and regional variability in mucus composition and goblet cell density [15,31,32]. It is therefore plausible that the enlargement of goblet cells and the shift in goblet-cell-to-enterocyte ratios that we report may reflect an adaptive adjustment of the mucus layer to chronic apelin exposure in the developing gut, but this remains an assumption.

The changes in MP and SP ganglion size and shape indicate that apelin-13 is associated with structural remodeling within the enteric plexuses during postnatal gut development. This fits with evidence that the apelin receptor is present on enteric neurons, as APJ has been detected on myenteric ChAT-positive and nNOS-positive neurons, and apelin can directly modulate duodenal contractions through an enteric pathway [20]. Functional studies further show that apelin-13 alters gastrointestinal motor activity through APJ-dependent signaling, both under basal conditions and during stress, supporting a physiologic link between apelin and enteric neural control of motility [33,34,35]. In our material, MP ganglia were generally larger in proximal and middle segments but smaller in the distal jejunum, whereas SP ganglia showed a proximal and ileal increase with reductions through much of the jejunum. A plausible interpretation is that chronic luminal apelin shifts the balance of excitatory and inhibitory enteric circuits in a region-specific way, potentially mirroring the known proximal dominance of nutrient sensing and apelin signaling in early life [12,14]. However, we did not assess neuronal subtypes, neurotransmitters, or motility, so any inference about excitatory or inhibitory balance remains highly speculative. Another, not mutually exclusive, possibility is that apelin-13 influences the normal maturational remodeling of enteric ganglia, since adolescence and early postnatal life are periods of rapid anatomical and chemical reorganization of enteric neurons and glia [36]. Unfortunately, without direct motility testing, it remains uncertain how the observed morphometric shifts translate into function. Taken together with prior demonstrations that apelin acts on defined enteric neuronal populations, our purely morphological data are compatible with a role of the apelin-APJ system in shaping enteric plexus structure during intestinal development, but functional implications will require dedicated motility and electrophysiological studies.

The shifts in ghrelin and leptin immunoreactivity add an endocrine dimension to the structural changes described above and indicate altered local distribution of these appetite-related peptides in a region-specific manner. Our finding that ghrelin-positive area increased in most regions is qualitatively in line with reports that repeated apelin-13 can raise circulating ghrelin and increase food intake in adult rats, although we did not measure circulating levels or feeding behavior in this study [23,37]. A similar orexigenic profile has been reported after central apelin-13 delivery, where increased feeding was interpreted as a hypothalamic effect, indicating that apelin can engage appetite circuits at multiple levels [12,38]. In our study, the rise in ghrelin immunoreactivity occurred primarily in the stomach and in most intestinal segments, i.e., in regions critical for the maturation of the ghrelin axis [39], with no change in the middle jejunum. Research by Valverde Piedra et al. demonstrated that interference with the ghrelin axis alters the proliferation and maturation of the intestinal epithelium in young rats, indicating that gastrointestinal hormones play a key role in the developmental regulation of gastrointestinal endocrine cells [40]. Since ghrelin-producing cells are densest in the stomach but are present throughout the small intestine, including the jejunum [41,42], it is plausible that apelin-13 either enhances ghrelin cell activity directly via APJ signaling or indirectly by altering mucosal maturation and nutrient sensing [43]. These results suggest that apelin-13 may function as a key hormone modulator of the local ghrelin system during the suckling period. It is also worth noting that Diab et al. reported reduced ghrelin in gastric secretion after apelin-13, together with changes in acid output [26], which differs from our tissue-based findings. The discrepancy may stem from major design differences: their work used adult rats, assessed luminal ghrelin content rather than mucosal immunoreactivity, and included acute systemic dosing, whereas our model involves unweaned animals with chronic intragastric exposure. Leptin immunoreactivity showed a more heterogeneous pattern, increasing in the stomach and distal jejunum but decreasing in the duodenum and ileum. This arrangement is biologically plausible, as gastric leptin is produced by chief cells as well as endocrine cells and is released into both the lumen and circulation, where it contributes to satiety signaling and modulation of gastric emptying [44]. The parallel immunoreactivity of ghrelin and leptin in the stomach, despite the well-established functional antagonism between these hormones, suggests that apelin-13 exerts its effects at a relatively early stage of endocrine cell differentiation, preceding the maturation of canonical ghrelin-leptin regulatory interactions [39]. This interpretation is further supported by the markedly high expression of the APJ receptor in the stomach of young rats, which renders this tissue particularly responsive to apelin during the early postnatal period. Collectively, these observations underscore the developmental and pro-proliferative role of apelin in shaping the endocrine compartment of the immature stomach [28]. Segmental differences in intestinal leptin signaling are expected during development because leptin receptors and leptin-responsive pathways mature unevenly along the gut and are sensitive to local nutritional cues [45,46]. In adult rats, apelin-13 can increase systemic leptin at higher doses [23], so the rise we observed in the stomach and distal jejunum is consistent with a positive apelin–leptin association. The opposite changes in the duodenum and ileum may reflect regional differences in APJ–leptin crosstalk or compensatory adjustments within the orexigenic–anorexigenic network, but our data cannot distinguish between altered leptin synthesis, storage, and release. Taken together, the ghrelin-dominant increase alongside selective up- or down-regulation of leptin suggests that apelin-13 locally remodels the ghrelin–leptin axis, potentially shaping appetite-related signaling locally in the developing gut, yet the functional consequence for feeding behavior will need direct testing in future work. It should be noted that our measure of percentage immunopositive area cannot discriminate between an increased number of ghrelin- or leptin-producing cells and increased peptide content per cell, or a combination of both. Our conclusions are therefore limited to regional changes in the density of immunohistochemical labeling rather than direct statements about cell counts or secretory output.

## 5. Conclusions

In conclusion, chronic intragastric apelin-13 during early postnatal life was associated with segment-specific alterations in gastric and intestinal wall morphology, enterocyte and goblet cell profiles, enteric plexus structure, and local ghrelin and leptin immunoreactivity. These findings provide a detailed morphological and immunohistochemical description of how the immature gastrointestinal tract responds to apelin-13 and form a basis for hypotheses about potential consequences for motility, barrier function, and appetite regulation. Future work that combines similar structural approaches with direct functional measurements will be required to define how these changes affect gastrointestinal performance and systemic energy balance.

## Figures and Tables

**Table 1:** Primary and secondary antibodies used in this study.

Antibody	Host	Code	Dilution	Source
Anti-Ghrelin	Rabbit	Ab134978	1:100	Abcam
Anti-Leptin	Rabbit	Ab117751	1:100	Abcam
Anti-Neurofilament	Rabbit	N4142-.2ML	1:40	Sigma-Aldrich
Anti-mouse/rabbit	Goat	DPVB-HRP	RTU ^1^	ImmunoLogic

^1^ RTU = Ready to Use.

**Table 2:** Percentage of ghrelin- and leptin-immunopositive mucosal area in the stomach and small intestinal segments of control and apelin-treated unweaned Wistar rats. Values are presented as mean ± SEM (*n* = 6 per group). Asterisks indicate significant differences between control and apelin groups within a given segment (* *p* < 0.05, *** *p* < 0.001).

Segment	Ghrelin		Leptin	
	Control	Apelin	Control	Apelin
Stomach	6.6 ± 0.6	10.9 ± 1.2 ***	7.8 ± 0.6	14.6 ± 1.2 ***
Duodenum	3.2 ± 0.4	4.9 ± 0.5 ***	0.17 ± 0.013	0.12 ± 0.020 ***
Jejunum 25%	4.0 ± 0.4	7.4 ± 0.8 ***	0.07 ± 0.008	0.06 ± 0.006
Jejunum 50%	3.7 ± 0.6	3.8 ± 0.3	0.05 ± 0.003	0.06 ± 0.007
Jejunum 75%	2.3 ± 0.2	8.7 ± 0.4 ***	0.31 ± 0.019	0.33 ± 0.021 *
Ileum	0.7 ± 0.1	4.6 ± 0.4 ***	0.16 ± 0.027	0.09 ± 0.005 ***

## Data Availability

The data that support the findings of this study are available on request from the corresponding authors.

## Abbreviations

The following abbreviations are used in this manuscript:

CNS	Central Nervous System
ENS	Enteric Nervous System
MP	Myenteric Plexus
SP	Submucosal Plexus
H&E	Haematoxylin and Eosin
AB-PAS	Alcian Blue and Periodic Acid-Schiff
